# Impact of ITS-Based Sequencing on Antifungal Treatment of Patients with Suspected Invasive Fungal Infections

**DOI:** 10.3390/jof6020043

**Published:** 2020-03-27

**Authors:** Sara Guenter, Gregor Gorkiewicz, Bettina Halwachs, Karl Kashofer, Andrea Thueringer, Phillip Wurm, Ines Zollner-Schwetz, Thomas Valentin, Juergen Prattes, Stefanie Wunsch, Elisabeth Ullrich, Christoph Zurl, Martin Hoenigl, Robert Krause

**Affiliations:** 1 Section of Infectious Diseases and Tropical Medicine, Department of Internal Medicine, Medical University of Graz, 8010 Graz, Austria; sara.michele.guenter@gmail.com (S.G.); ines.schwetz@medunigraz.at (I.Z.-S.); thomas.valentin@klinikum-graz.at (T.V.); juergen.prattes@medunigraz.at (J.P.); stefanie.wunsch@medunigraz.at (S.W.); elisabeth.ullrich@medunigraz.at (E.U.); christoph.zurl@medunigraz.at (C.Z.); mhoenigl@health.ucsd.edu (M.H.); 2 Diagnostic and Research Institute of Pathology, Medical University of Graz, 8010 Graz, Austria; gregor.gorkiewicz@medunigraz.at (G.G.); bettina.halwachs@medunigraz.at (B.H.); karl.kashofer@medunigraz.at (K.K.); andrea.thueringer@medunigraz.at (A.T.); philipp.wurm@medunigraz.at (P.W.); 3 BioTechMed-Graz, 8010 Graz, Austria; 4 Institute of Hygiene, Microbiology and Environmental Medicine, Medical University of Graz, 8010 Graz, Austria; 5 Division of General Paediatrics, Department of Paediatrics and Adolescent Medicine, Medical University of Graz, 8010 Graz, Austria; 6 Division of Infectious Diseases and Global Public Health, Department of Medicine, University of California San Diego, San Diego, CA 92093, USA

**Keywords:** invasive fungal infections, ITS-sequencing, antifungal therapy

## Abstract

Molecular techniques including the sequencing of fungal-specific DNA targets are increasingly used in the diagnosis of suspected invasive fungal infections. In contrast to established biomarkers like galactomannan or 1-3-β-d-glucan, the clinical impact of these methods remains unknown. We retrospectively investigated the impact of ITS1-sequencing on antifungal treatment strategies in 71 patients (81 samples) with suspected invasive fungal infections. ITS-sequencing either confirmed already ongoing antifungal therapy (19/71 patients, 27%), led to a change in antifungal therapy (11/71, 15%) or supported the decision to withhold antifungal treatment (34/71, 48%) (in seven of 71 patients, ITS-sequencing results were obtained postmortem). ITS-sequencing results led to a change in antifungal therapy in a relevant proportion of patients, while it confirmed therapeutic strategies in the majority. Therefore, ITS-sequencing was a useful adjunct to other fungal diagnostic measures in our cohort.

## 1. Introduction

Fungal infections are increasing worldwide [1,2,3]. The survival rate of invasive fungal infections (IFIs) is associated with the early initiation of antifungal therapy, since a delay in the administration of antifungals significantly impacts mortality [4,5]. In clinical practice, the diagnosis of fungal infection is primarily based on direct microscopic examination of clinical samples, biomarker testing, histopathology and cultures of blood or tissue. Importantly, the sensitivity of culture-based methods is usually below 50%, and culture-based methods may fail to diagnose invasive fungal infections, particularly during their early stages [6,7]. Other diagnostic measures like specific PCR methods lack sensitivity and specificity [8,9]. The newly established T2 magnetic resonance method showed promising diagnostic performance for *Candida*-spiked blood cultures. However, recently published data from real life settings showed limited clinical value, as the T2 assay gave a negative result in some patients with culture-proven candidemia [10,11,12]. Biomarker testing using 1-3-β-d-glucan (BDG), galactomannan (GM) or tetra (1→5)-d-galactofuranoside (*Aspergillus* lateral flow test) improved diagnostic workup of patients with IFIs [13,14,15]. The modification of BDG testing allowed the rapid assessment of *Candida* colonization or invasive candidiasis due to the high negative predictive value of this “panfungal” antigen test [16]. However, BDG is false positive on several occasions, whereas the sensitivity of *Aspergillus*-specific antigen tests is limited in patients receiving mold active prophylaxis [6,17,18]. Additionally, to date, no clinical useful biomarker test is available for the diagnosis of *Mucorales* infections [19].

The PCR of internal transcribed spacer ITS-1 and -2-regions with subsequent sequencing offers identification of fungi causing IFIs [9,20,21]. Human samples undergoing ITS PCR and sequencing are usually obtained by invasive procedures like bronchoscopy or transthoracic puncture, or even by surgical resection of infected tissue. Molecular identification methods including nested PCRs, or the sequencing of ITS, 18S or other targets were recently recommended in the global guidelines for the diagnosis and management of mucormycosis [19]. Although sequencing methods are actually available in clinical routine, in some centers the clinical impact of this new technique with regards to antifungal therapy (initiation, confirmation, change in or withholding of antifungal treatment) is unknown. Recently, long-read sequencing was used for the detection and confirmation of *Pneumocystis* pneumonia cases, but the small sample size limited the assessment of clinical consequences of this promising new technique [22].

The aim of this retrospective study was to investigate the impact of ITS-sequencing on antifungal treatment of patients with suspected IFIs.

## 2. Materials and Methods

### 2.1. Patients

At the Medical University of Graz, Austria, ITS-sequencing is available for routine diagnostic work in cases with suspected IFIs. The decision of whether or not to perform ITS-sequencing is up to the treating physicians. ITS-sequencing is performed at the Institute of Pathology at the Medical University of Graz and results are stored in the local database. All patients who received ITS-sequencing for suspected IFI from any specimens during January 2015 (the implementation of ITS-sequencing at the Medical University of Graz) and March 2019 were eligible for the study. The clinical data of patients were extracted from computerized clinical databases, medical records and handwritten charts. IFIs were categorized as possible, probable or proven IFI cases based on recent recommendations and the utilization of standard microbiological/histological investigations including cultures, histopathology and biomarkers [23,24,25]. Sample preparation, ITS PCR and sequencing has been performed as described previously and implemented in the Microbiome/Mycobiome working group at the Institute of Pathology, Medical University of Graz [20]. The clinical impact of ITS-sequencing results was assessed by evaluation of the influence of ITS-sequencing on therapeutic procedures (e.g., the administration of new antifungal drugs; continuation of ongoing antifungal treatment; withdrawal of antifungal drugs). In addition, ITS-sequencing results were compared to conventional microbiological investigations (fungal culture) The data were extracted from the electronical databases or handwritten charts. Specimens of fungal cultures, histopathology, biomarker testing and ITS-sequencing were collected in the same procedure (e.g., bronchoscopy, biopsy) and under the responsibility of the treating physician. Fungal cultures were performed in local microbiology laboratories with material obtained in addition to ITS-sequencing samples during the same sampling procedure. Imaging procedures included CT, MRI, PET CT and leukocyte scintigraphy scans and were analyzed by radiologists during clinical routine. The study protocol was approved by the local ethics committee, Medical University of Graz, (protocol number 31-383 ex 18/19) including a waiver of informed consents, as the data evaluation was collected retrospectively.

### 2.2. DNA Isolation and PCR Amplification

Fungal DNA was extracted with the Maxwell RSC Blood DNA Kit (Promega, Mannheim, Germany) according to the manufacturer’s instructions with slight modifications. Using the Lysis Buffer, stool samples were homogenized on a MagNA Lyser Instrument using MagNA Lyser Green Beads (Roche Diagnostics GmbH, Mannheim, Germany) (6000 rpm for 30 sec). After homogenization, samples were treated with 2.5 mg/mL Lysozyme (Roth GmbH, Karlsruhe, Germany) for 30 min at 37 °C followed by digestion with 1 mg/mL Proteinase K for 60 min at 56 °C. Enzyme activity was inactivated for 10 min at 95 °C and 600 μL of lysate was used for the DNA isolation in the Maxwell RSC. DNA concentration was measured by Picogreen fluorescence. The variable ITS region of the fungal 5.8S rRNA gene was amplified with PCR from 20 ng DNA using oligonucleotide primers ITS1_fwd: TCCGTAGGTGAACCTGCGG and ITS2_rev: GCTGCGTTCTTCATCGATGC. This ITS rDNA region was chosen, since it gives a robust taxonomic classification and has been shown to be suitable for community clustering. The ITS1 region was amplified with the Mastermix 16S Complete PCR Kit (Molzym, Bremen, Germany) according to the manufacturer’s instructions using a 0.4 µM final concentration of primers and 57 °C annealing temperature for 25 cycles. The first PCR reaction product was subjected to a second round of PCR with primers fusing the ITS primer sequence to the A, and P adapters necessary for Ion Torrent sequencing, while additionally including a molecular barcode sequence to allow the multiplexing of up to 96 samples simultaneously. PCR products were subjected to agarose gel electrophoresis, and the band of the expected length (350 nt) was excised from the gel and purified using the QiaQick (Qiagen, Hilden, Germany) gel extraction system. The DNA concentration of the final PCR product was measured by Picogreen fluorescence.

### 2.3. Sequencing

Amplicons from up to 60 samples were pooled equimolarly and subjected to emulsion PCR using the Ion 530 Chef Kit and the 400 bp workflow according to the manufacturer’s protocols. After emulsion PCR, the beads are loaded onto Ion Torrent 530 chips for sequencing. Sequencing reactions were performed on Ion Torrent S5XL using the Ion 400 bp Sequencing Kit running for 1000 flows (all reagents from Thermo Fisher Scientific, Waltham, MA, USA). Sequences were split by barcode and transferred to the Torrent Suite server. Unmapped bam files were used as input for bioinformatics.

### 2.4. Bioinformatics and Phylogenic Analysis

All sequences were initially trimmed by a sliding window quality filter with a width of 20 nt and a cutoff of Q20. Reads shorter than 100 nucleotides and reads mapping to the human genome were removed using DeconSeq [26]. The resulting reads were subjected to error correction using the Acacia tool [27], leading to an error correction of 10–20% of reads. Subsequently, PCR chimeras were removed by the usearch algorithm in de novo and reference-based settings [28]. The final sequence files were then analyzed by QIIME 1.8 workflow scripts [29]. An OTU search was performed using the parallel_pick_open_reference_otus workflow script and the Unite 12_11 reference database.

### 2.5. Statistical Analysis and Visualization

OTUs were visualized as OTU tables, bar charts and PCOA plots using the QIIME core microbiome script. Additionally, the groupings supplied in the mapping file were tested for statistical significance using the QIIME implementation of the Adonis test and the significance of individual fungal strains was determined by Kruskal–Wallis test. LEfSe analysis was performed to detect statistically relevant strains [30].

## 3. Results

A total of 71 patients (median age 54 years, 26 female) with a total of 81 samples (four patients had two samples and three had three samples) undergoing ITS-sequencing were included in the study. Demographics including underlying diseases are shown in Table 1. The type and number of 81 tissue or body fluid samples used for ITS PCR and sequencing are shown in a footnote of Table 1.

**Figure 1:**
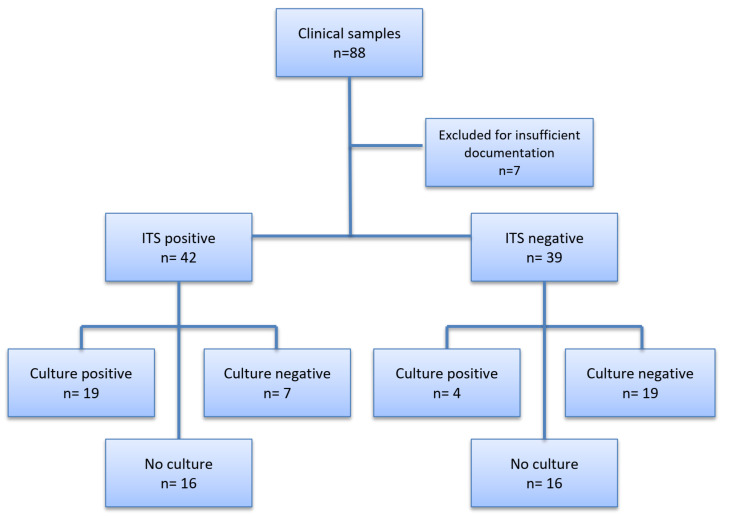
Flowchart of ITS-sequencing and conventional cultures. *n* = numbers.

ITS PCR and sequencing were ordered by infectious diseases consultants for 29 (36%) of samples, by pathologists for 16 (20%), and by others for 36 (44%) of samples. Out of the 69/71 patients with sufficient data for IFI classification, nine (13%) had proven, 10 (15%) had probable, five (7%) had possible and 45 (65%) did not meet EORTC/MSG-criteria for IFI at the timepoint of sampling. In two patients, data were insufficient for the classification of IFI. A total of 16 (23%) patients died during hospitalization, including two IFI-related and 14 IFI-unrelated deaths. Samples from the lower respiratory tract (lung tissue, *n* = 20; BAL, *n* = 8) were most commonly investigated, followed by samples from the skin (*n* = 9), eyes (*n* = 5), liver (*n* = 4), and others (*n* = 35). A fungal pathogen was detected by ITS-sequencing in 42 samples (52%), whereas no fungal pathogen was obtained in the remaining 39 samples. The most common fungi obtained by ITS-sequencing were *Candida* sp. (36%) (including *Candida albicans, C. krusei, C. guilliermondii, Candida parapsilosis*), *Aspergillus* sp. (24%), *Cladosporium* sp. (17%), *Fusarium* sp. (5%), and Mucorales (5%) (*Rhizopus microspores, Actinomucor elegans*). Mixed fungal genera were found in 16% of samples. Out of 42 samples with positive ITS-sequencing, 19 (45%) showed also fungal growth in cultures, while seven cultures did not yield fungal growth, and cultures were not performed in the remaining 16 of these 42 samples. In 39 samples with negative ITS-sequencing, four showed positive cultures, 19 negative cultures, and culture were missing in 16 of these 39 samples. Therefore, concurrent culture and ITS-sequencing was performed with 49/81 samples (60%). In 19 samples, fungal culture and ITS-sequencing were positive with discordant identification in five of these 19 (26%) samples. In the remaining 23 samples, ITS-sequencing was positive, whereas fungal culture was negative in seven samples (fungi identified by ITS included *Cladosporium sp., Aspergillus sp.* and *Candida sp*.) or not performed in 16 samples. In four samples, fungal cultures were positive, whereas ITS-sequencing was negative. Cultures were not ordered or performed with 32/81 (40%) samples (Figure 1). The reasons for omitting the cultures for these particular cases were not specified in patient charts.

Overall, ITS-sequencing results informed antifungal treatment strategy in 64/71 (90%) of patients. In 16 patients (23%) with positive ITS-sequencing results and missing culture, therapy was modified in 11 patients (69%), and therapy was considered as an already targeted treatment addressing the identified fungus in 2/16 patients (13%). In 60 patients (85%), the antifungal treatment strategy remained unchanged after the receipt of ITS-sequencing due to the following reasons: ITS-sequencing remained negative, and there was no need for antifungal therapy in 26/60 patients (43%); ITS-sequencing result was interpreted as contamination in 9/60 (15%); adequate antifungal therapy was already ongoing without need for modification in 17/60 (28%); and ITS-sequencing results were obtained postmortem in 7/60 (12%). In one patient with a positive ITS-sequencing result from a corneal sample, local antifungal therapy was initiated but not systemic antifungal treatment.

## 4. Discussion

In our study involving 81 tissue or body fluid samples from 71 patients ITS-sequencing enabled identification of a fungal pathogen in 52% of cases with *Candida* sp. (36%), *Aspergillus* sp. (24%), *Cladosporium* sp. (17%), *Fusarium* sp. (5%) and Mucorales (5%) as the leading pathogens. In 90% of patients, the ITS-sequencing results came back when patients were still alive, and informed therapeutic strategies. Despite advances in time to results and in the spectrum of microorganisms covered by new sequencing technologies, many challenges were previously discussed, including DNA quality, a low DNA to host ratio, lack of quality-controlled reference databases, contamination issues and bioinformatic tools [20,31,32,33]. In addition, compared to well established fungal biomarkers (e.g., BDG, GM), the clinical impact of sequencing technologies in terms of clinical management has rarely been investigated. In this project, we demonstrated that modern sequencing technologies had an impact on decision making with regard to antifungal therapy. In seven of 28 (25%) cases, ITS-sequencing results supported the assessment of, e.g., unclear lung infiltrations in patients at risk for IFIs and contributed to clarification of the etiology. In these cases, ITS-sequencing confirmed ongoing antifungal therapy or informed a change in antifungal treatment strategy. Besides well-established fungal diagnostic biomarkers and conventional diagnostic measures, ITS-sequencing can therefore be considered as an additional laboratory tool useful for antifungal stewardship activities.

As a limitation, only 60% of patients in our cohort had concurrent culture and ITS-sequencing of their samples. The reasons for missing cultures in 40% of samples remained unclear, but may be due to local procedures, as cultures and ITS-sequencing were performed in two distant laboratories (cultures in the microbiological laboratory and ITS-sequencing in the pathology laboratory). Clinicians therefore had to send two adjacent samples to each of the labs. In 26% of samples undergoing combined ITS-sequencing and cultures, the investigations showed discordant results. ITS-sequencing might therefore provide additional information in patients with difficult-to-culture fungi or in patients already receiving antifungal treatment prior to sampling, which might influence fungal growth. In seven patients, ITS-sequencing results were obtained postmortem. Time-to-result is a critical issue for every diagnostic tool especially in rapidly progressive fungal diseases like mucormycosis [19]. As no fungal biomarker can be used for the anticipation of mucormycosis, faster sequencing technologies of tissues or other samples may prospectively be useful in the earlier diagnosis of this often fatal disease [19].

Based on the data presented, ITS-sequencing has been established at our center as a routine investigation in addition to all the other diagnostic tools, like culture, biomarker testing including recently released *Aspergillus* lateral flow device tests and PCRs. In some studies, the sequencing of amplicons of 18S or ITS2 genes outperformed the results created by using ITS1 targets, as used in our study [34,35].

In conclusion, our study found that ITS-sequencing, despite a long turnaround time, informed antifungal treatment strategies in 90% of patients. ITS-sequencing confirmed ongoing antifungal therapy (19/71 patients, 27%), led to a change in antifungal therapy (11/71, 15%) or supported the decision to withhold antifungal treatment (34/71, 48%). Owing to the current difficulties in elucidating the fungal etiology and subsequently selecting the correct antifungal treatment, we need further data for modern molecular-based technologies, including metagenomic shotgun long-read sequencing, for the identification of pathogenic fungal species in patients with IFIs.

## Figures and Tables

**Table 1:** Demographics of 71 patients, providing 81 samples * for ITS-sequencing.

Type		No. (%)	Age, Years, Median (IQR)
Female sex, No. (%)		26 (37%)	54 (46–69)
Invasive fungal infections	Possible	5 (7%)	
	Probable	10 (15%)	
	Proven **	9 (13%)	
Type of fungal infection	Aspergillosis	14 (20%)	
	Mycormycosis	3 (4%)	
	Others	52 (73%)	
Underlying diseases	Solid cancer	17 (24%)	
	Malignant hematological disease	12 (17%)	
	Congenital disorder	7 (10%)	
	Diabetes mellitus	5 (7%)	
	Solid organ transplantation	5 (7%)	
	Stem cell transplantation	1 (1%)	
	Miscellaneous underlying diseases not considered as IFI risk factors	25 (35%)	
No underlying diseases		13 (18%)	

IFI = invasive fungal infection. * = The specimen included: lung tissue (*n* = 21), skin (9), bronchoalveolar lavage fluid (8), eye specimen (5), liver tissue (4), sinus tissue or swab (4), pleural fluid (4), stool (3), liquor (3), sputum (2) and others (18), including two aortic valves, two maxillary sinuses specimen, two tissue samples obtained from the middle ear, two lymph nodes, two bone samples, one retroperitoneal tissue sample, one otorrhoea swab, one omentum tissue sample, one lumbar vertebrae sample, one brain abscess aspirate, one pericardium tissue sample and one intervertebral disc tissue sample. ** = proven cases included invasive Aspergillosis, invasive Candidiasis, invasive *Cladosporium* infection.
